# Quantum-Like Bayesian Networks for Modeling Decision Making

**DOI:** 10.3389/fpsyg.2016.00011

**Published:** 2016-01-26

**Authors:** Catarina Moreira, Andreas Wichert

**Affiliations:** Department of Computer Science, Instituto Superior Técnico, University of Lisbon, INESC-IDLisbon, Portugal

**Keywords:** Bayesian networks, decision making, quantum probability, quantum cognition, sure thing principle

## Abstract

In this work, we explore an alternative quantum structure to perform quantum probabilistic inferences to accommodate the paradoxical findings of the Sure Thing Principle. We propose a Quantum-Like Bayesian Network, which consists in replacing classical probabilities by quantum probability amplitudes. However, since this approach suffers from the problem of exponential growth of quantum parameters, we also propose a similarity heuristic that automatically fits quantum parameters through vector similarities. This makes the proposed model general and predictive in contrast to the current state of the art models, which cannot be generalized for more complex decision scenarios and that only provide an explanatory nature for the observed paradoxes. In the end, the model that we propose consists in a nonparametric method for estimating inference effects from a statistical point of view. It is a statistical model that is simpler than the previous quantum dynamic and quantum-like models proposed in the literature. We tested the proposed network with several empirical data from the literature, mainly from the Prisoner's Dilemma game and the Two Stage Gambling game. The results obtained show that the proposed quantum Bayesian Network is a general method that can accommodate violations of the laws of classical probability theory and make accurate predictions regarding human decision-making in these scenarios.

## 1. Introduction

The present work proposes a new model to make predictions in paradoxical situations where the Sure Thing Principle is being violated. The Sure Thing Principle (Savage, 1954) is a fundamental principle in economics and probability theory and states that if one prefers action *A* over *B* under state of the world *X*, and if one also prefers *A* over *B* under the complementary state of the world, ¬ *X*, then one should always prefer action *A* over *B* even when the state of the world is unspecified. Several experiments have shown that people violate this principle in decisions under uncertainty, leading to paradoxical results and violations of the classical law of total probability (Tversky and Kahnenman, 1974; Tversky and Kahneman, 1983; Tversky and Shafir, 1992; Aerts et al., 2004; Birnbaum, 2008).

### 1.1. Motivation

More recently, cognitive scientists have turned to quantum probability theory in order to accommodate these paradoxical findings. Although many models have been proposed in the literature, most of them cannot be considered predictive. Most of these models require a set of quantum parameters to be fitted and, so far, the only way these models have to fit the parameters is to use the final outcome of the experiment to set the parameters in order to explain that outcome. Moreover, these models cannot scale to more complex decision scenarios, because the number of parameters is exponentially large (Khrennikov, 2003a, 2004, 2006) or because of computational constraints in the computation of very large unitary operators (Busemeyer et al., 2006b, 2009; Pothos and Busemeyer, 2009).

### 1.2. Contributions

For these reasons, in this work, we propose a network structure framework that can easily scale to more complex decision scenarios. In other words, we propose a quantum-like Bayesian Network formalism, which consists in replacing classical probabilities by quantum probability amplitudes. However, since this approach also suffers from the problem of exponential growth of quantum parameters that need to be fit, we also propose a similarity heuristic (Shah and Oppenheimer, 2008) that automatically computes this exponential number of quantum parameters through vector similarities. A Bayesian Network can be understood as an acyclic directed graph, in which each node represents a random variable and each edge represents a direct causal influence from the source node to the target node (conditional dependence).

In this article, we will address the problem of violations to the Sure Thing Principle by examining two major problems in which these violations were verified: the Prisoner's Dilemma game and the Two Stage Gambling game. These violations were initially reported by Tversky and Shafir (1992) and later simulated in several works in the literature that also reported similar results (Li and Taplin, 2002; Busemeyer et al., 2006a; Hristova and Grinberg, 2008). We will show how the current classical models fail to explain the paradoxical findings implied in the violations of the Sure Thing Principle and we will make a more deep discussion about the drawbacks of the most representative quantum-like models in the literature.

### 1.3. Research questions

With the present work, we intend to address the following research questions. An answer to these questions is given in Section 8.

1. Why do we need another quantum-like model to explain violations to the Sure Thing Principle?
2. What is the advantage of the proposed approach? How can it make a difference toward the current well-established quantum models that have been proposed in the literature?

## 2. Violations of the sure thing principle

In this section, we present two experiments from the literature, in which it was observed violations to the Sure Thing Principle and consequently to the laws of classical probability theory and logic. The two experiments are the Prisoner's Dilemma game and the Two Stage Gambling game.

### 2.1. The Prisoner's Dilemma game

The Prisoner's Dilemma game corresponds to an example of the violation of the Sure Thing Principle. In this game, there are two prisoners who are in separate solitary confinements with no means of speaking to or exchanging messages with the other. The police offer each prisoner an agreement: each prisoner is given the opportunity either to betray the other (*Defect*), by testifying that the other committed the crime, or to *Cooperate* with the other by remaining silent.

In order to test the veracity of the Sure Thing Principle under the Prisoner's Dilemma game, an experiment was made in which three conditions were tested:

- Participants were informed that the other participant chose to *Defect*.
- Participants were informed that the other participant chose to *cooperate*.
- Participants had no information about the other participant's decision.

Table 1 summarizes the results of several works of the literature, which have performed this experiment. Note that all entries of Table 1 show a violation of the law of total probability. According to the total law of probability, it is expected that:

$$\begin{array}{l} Pr\left(P_{2}=Defect|P_{1}=Defect\right)\geq Pr\left(P_{2}=Defect\right)\\ \;\geq Pr\left(P_{2}=Defect|P_{1}=Cooperate\right) \end{array}$$

**Table 1:** **Works of the literature reporting the probability of a player choosing to *Defect* under several conditions for the Prisoner's Dilemma Game: when the action of the second player is known to be *Defect* (Known to Defect), when the action of the second player is known to be *Cooperate* (Known to Collaborate), and when the action of the second player is not known (Unknown)**.

**Literature**	**Known to Defect**	**Known to Collaborate**	**Unknown**	**Classical probability**
Shafir and Tversky, 1992	0.9700	0.8400	0.6300	0.9050
Croson, 1999^a^	0.6700	0.3200	0.3000	0.4950
Li and Taplin, 2002^b^	0.8200	0.7700	0.7200	0.7950
Busemeyer et al., 2006a	0.9100	0.8400	0.6600	0.8750
Hristova and Grinberg, 2008	0.9700	0.9300	0.8800	0.9500
Average	0.8700	0.7400	0.6400	0.8050

a *corresponds to the average of the results reported in the first two payoff matrices of the work of Croson (1999)*.

b *corresponds to the average of all seven experiments reported in the work of Li and Taplin (2002)*.

Note that, *Pr*(*P*_2_ = *Defect* | *P*_1_ = *Defect*) corresponds to the probability of the second player choosing the *Defect* action given that he knows that the first player chose to *Defect*. In Table 1 this corresponds to the entry *Known to Defect*. In the same way, *Pr*(*P*_2_ = *Defect* | *P*_1_ = *Cooperate*) corresponds to the entry *Known to Collaborate*. The observed probability during the experiments concerned with player 2 choosing to *Defect*, *Pr*(*P*2 = *Defect*), corresponds to the entry *unknown* of Table 1, since there is no evidence about the first player's actions. Finally, the entry *Classical Probability* corresponds to the classical probability *Pr*(*P*_2_ = *Defect*), which is computed through the law of total probability:

$$\begin{array}{l} Pr\left(P_{2}=Defect\right)=Pr\left(P_{1}=Defect\right)\cdot Pr(P_{2}=Defect|\\ \;P_{1}=Defect)+\\ \;+Pr(P_{1}=Cooperate)\cdot Pr(P_{2}=Defect|P_{1}\\ \;=Cooperate) \end{array}$$

### 2.2. The two stage gambling game

The Two Stage Gambling game is another game that shows violations of the Sure Thing Principle. In this game, participants were asked at each stage to make the decision of whether or not to play a gamble that has an equal chance of winning $200 or losing $100. Three conditions were verified:

1. Participants were informed if they had won the first gamble;
2. Participants were informed if they had lost the first gamble;
3. Participants did not know the outcome of the first gamble;

The overall results revealed that participants who knew that they won the first gamble, decided to play again. Participants who knew that they lost the first gamble, also decided to play again. Through Savage's Sure Thing Principle, it was expected that the participants would choose to play again, even if they did not know the outcome of the first gamble. However, the results obtained revealed something different. If the participants did not know the outcome of the first gamble, then many of them decided not to play the second one.

We conclude this section by clarifying why we will only validated the proposed quantum-like Bayesian Network in small decision problems (such as the Prisoner's Dilema and the Two Stage Gambling Game), since we are defending a general quantum-like structure that is able to deal with complex decision scenarios. We used small decision scenarios, because we cannot find literature showing violations to the Sure Thing Principle for more complex decision scenarios. Actually, after performing some research, we believe that the violations of the Sure Thing Principle tend to diminish with the complexity of the decision scenario. Imagine for instance a three stage gambling game. It will be very hard to find significant data that shows a player wishing to play the last gamble, given that he has lost the two previous gambles. Table 2 shows the results obtained in several works of the literature.

**Table 2:** **Works of the literature reporting the probability of a player choosing to make a second gamble under several conditions for the Two Stage Gambling Game: when the outcome of the first gamble is known to be *Lose* (Known to Lose), when the outcome of the first gamble is known to be *Win* (Known to Win), and when the outcome of the first gamble is not known (Unknown)**.

**Literature**	**Known to Win**	**Known to Lose**	**Unknown**	**Classical probability**
Tversky and Shafir, 1992	0.69	0.58	0.37	0.6350
Kuhberger et al., 2001	0.72	0.47	0.48	0.5950
Lambdin and Burdsal, 2007	0.63	0.45	0.41	0.5400
Average	0.68	0.50	0.42	0.5900

## 3. Violation of the sure thing principle: Classical approaches

There are many classical approaches that could be used to try to accommodate violations to the Sure Thing Principle. Two of these main models are the Classical Markov Models and the Classical Bayesian Networks. In this section, we will describe how these two models work and we will explain why they cannot be used to simulate violations to the Sure Thing Principle.

### 3.1. Classical Markov Model

A Markov Model can be generally defined as a stochastic probabilistic undirected graphical model that satisfies the Markov property. This means that the process evolves (and tries to perform a prediction) based only on the present state. The current state is independent of any past or future states. These probabilistic models are very useful to model systems that change states according to a transition matrix that specifies some probability distribution or some transition rules that depend solely on the current state.

The initial state is given by a vector, which contains the probabilities of each event occurring. This vector requires that the sum of these probabilities is one.

$$P_{I}=\left[\begin{array}{cccc} a_{0} & a_{1} & \ldots & a_{n} \end{array}\right]\cdot \frac{1}{\sum_{i}a_{i}}$$

The state transition is represented by a differential equation, which consists in the multiplication of this initial probability state *P*_*I*_ by a transition function *T*(*t*). This function is represented by a matrix containing positive real numbers and with the constraint that each row must sum to one (normalization axiom). In other words, this matrix represents the new probability distribution across all possible outcomes through some time period *t* (Pothos and Busemeyer, 2009).

(1) $$\frac{d}{dt}T(t)=K\cdot T(t)$$

The intensity matrix *K* corresponds to the problem's settings. For instance, for the Prisoner's Dilemma Game, it represents the payoffs of each player, in the Two Stage Gambling Game, it represents the rewards/losses that the player can have in each gamble. A solution to this equation is given by Equation 2, which allows one to construct a transition matrix for any time point from the fixed intensity matrix. In other words, the intensity matrix performs a transformation in the probabilities of the current state in order to favor a certain action in the decision problem.

(2) $$T(t)=e^{K.t}$$

In the end, we can compute the solution for the probability distribution over time by multiplying the transition matrix by the initial probability state.

(3) $$P_{F}(t)=e^{K.t}\cdot P_{I}(0)$$

In Equation 3, we do not need to perform any normalization in the end, because the operation in Equation 1 together with the intensity matrix *K* assure that the values computed are already probability values.

Since, in the end, the Markov Model has to obey to the rules of probability theory and set theory, even if we parameterize the intensity matrix *K*, we would find that there are no values that could explain the violations of the Sure Thing Principle without violating the laws of classical probability theory. Some studies have been proposed in the literature demonstrating that the classical Markov Model cannot accommodate violations to the Sure Thing Principle (Busemeyer et al., 2009; Pothos and Busemeyer, 2009).

### 3.2. Classical Bayesian Networks

A classical Bayesian Network can be defined by a directed acyclic graph structure in which each node represents a different random variable from a specific domain and each edge represents a direct influence from the source node to the target node. The graph represents independence relationships between variables and each node is associated with a conditional probability table, which specifies a distribution over the values of a node given each possible joint assignment of values of its parents. This idea of a node, depending directly from its parent nodes, is the core of Bayesian Networks. Once the values of the parents are known, no information relating directly or indirectly to its parents or other ancestors can influence the beliefs about it (Koller and Friedman, 2009).

A Bayesian Network can be understood as the representation of a full joint probability distribution through conditional independence statements. This way, a Bayesian Network can be used to answer any query about the domain by combining (adding) all relevant entries from the joint probability.

The full joint distribution (Russel and Norvig, 2010) of a Bayesian Network, where *X* is the list of variables, that is, the set of nodes of the Bayesian Network and is given by:

(4) $$Pr(X_{1},\ldots ,X_{n})=\prod_{i=1}^{n}Pr(X_{i}|Parents(X_{i}))$$

The formula for computing classical exact inferences on Bayesian Networks is based on the full joint distribution (Equation 4). Let *e* be the list of observed variables (nodes) and let *Y* be the remaining unobserved variables (nodes) in the network. For some query *X*, the inference is given by Equation 5. Note that, *Pr*(*X, e, y*) corresponds to the full joint probability distribution.

(5) $$Pr(X|e)=\alpha \left[\sum_{y\in Y}Pr(X,e,y)\right]$$

$$\text{Where}\;\alpha =\frac{1}{\sum_{x\in X}Pr_{c}(X=x,e)}$$

The summation is over all possible *y*, i.e., all possible combinations of values of the unobserved variables *y*. The α parameter, corresponds to the normalization factor for the distribution *Pr*(*X*|*e*) (Russel and Norvig, 2010). This normalization factor comes from some assumptions that are made in Bayes rule.

One might think that if we parameterize the Bayesian Network, it could be possible to explain the paradoxical findings of the Sure Thing Principle. This line of thought is legitimate, however one must take into account that in the end, the probabilistic inferences computed through the Bayesian Network must obey set theory and to the law of total probability. This means that, even if we parameterize the network, we could not find any closed form optimization that would accommodate violations to the Sure Thing Principle.

## 4. Violation of the sure thing principle: Quantum-like approaches

In this section, we introduce the most import quantum decision models that have been proposed in the literature that can accommodate the violations to the Sure Thing Principle. The models that we describe in this section are the following: the Quantum Dynamical Model (Section 4.1), the Quantum-Like Approach (Section 4.2), and the Quantum Prospect Decision Theory (Section 4.3).

### 4.1. The Quantum Dynamical Model

The Quantum Dynamical Model was originally proposed by Busemeyer (Busemeyer et al., 2009; Pothos and Busemeyer, 2009) and consists on a general framework that corresponds to a quantum version of a classical dynamical Markov model. The Quantum Dynamical Model takes into account time evolution. Quantum interference effects are also taken into account though a superposition of paths.

The initial belief state corresponds to a quantum state representing a superposition of the participant's beliefs in the form of a vector. The term ψ corresponds to a quantum probability amplitude.

(6) $$P_{I}=\left[\begin{array}{cccc} \psi_{0} & \psi_{1} & \ldots & \psi_{n} \end{array}\right]\cdot \frac{1}{\sum_{i}|\psi_{i}{|}^{2}}$$

Next, we need to create a unitary matrix. In quantum mechanics, a unitary matrix restricts the allowed evolution of quantum systems, ensuring that the sum of probabilities of all possible outcomes of any event is always 1. This means that the matrix must be orthonormal (the rows are mutually orthogonal unit vectors, as are the columns). In the Quantum Dynamical Model, this matrix encodes all state transitions that a person can experience while choosing a decision. The unitary matrix *U* is computed by a differential equation called Schrödinger's equation.

(7) $$\frac{\delta }{\delta t}U(t)=-i\cdot H\cdot U(t)$$

The parameter *t* corresponds to the time evolution. Under the Dynamical Quantum Model, this parameter is set to π∕2, corresponding to the average time that a participant takes to make a decision (approximately 2 s) (Pothos and Busemeyer, 2009). The matrix *H* is called the Hamiltonian matrix, which must be Hermitian in order to generate a unitary matrix.

(8) U(t)=exp(−i·H·t)

By multiplying the unitary matrix with the initial superposition belief state, one can compute the transition of the participants' beliefs at each time. The final vector *Q*_*F*_ represents the amplitude distribution across states after deliberation.

In the end, we can compute the solution for the probability distribution over time by multiplying the transition matrix by the initial probability state.

(9) $$Q_{F}=U\cdot Q_{i}=e^{-i\cdot H\cdot t}\cdot Q_{I}(0)$$

In Equation 9, we do not need to perform any normalization in the end, because the operation in Equation 8 together with the intensity matrix *H* assure that the values computed are in accordance with the normalization axiom.

### 4.2. The Quantum-Like Approach

The Quantum-Like Approach has its roots in contextual probabilities. This model was proposed by Khrennikov and corresponds to a general contextual probability space from which the classical and quantum probability models can be derived (Khrennikov, 2009b, 2010).

In the Quantum-Like Approach, the context relates to the circumstances that form the setting for an event in terms of which it can be fully understood, clarifying the meaning of the event. More specifically, it is a complex of conditions under which a measurement is performed. For instance, in domains outside of physics, such as cognitive science, one can have mental contexts. In social sciences, we can have a social context. And the same idea is applied to many other domains, such as economics, politics, game theory, biology, etc. (Khrennikov, 1999, 2001, 2003b, 2005a,b).

The Quantum-Like Approach corresponds to a contextual probabilistic model given by M = (C, O, π(O, C)). Where C is a set of contexts, O is the set of observables and π(O, C) corresponds to a probability distribution of some observables belonging to a specific context. Associated with a context, there are a set of observables. In quantum mechanics, an observable corresponds to a self-adjoint operator on a complex Hilbert Space. Under the Quantum-Like Approach, these observables correspond to the set of possible events with their respective values.

Let's assume, for a context *C* ∈ C, that there are two dichotomous observables *a, b* ∈ O, and each of these observables can take some values α ∈ *a* and β ∈ *b*, respectively.

The Quantum-Like Approach can be built from the general structure of the quantum law of total probability. The quantum law of total probability is very similar to the classical law of total probability, except that it uses complex amplitudes instead of real probability values. In order to obtain a probability value, the magnitude of the quantum amplitude must be squared Busemeyer and Bruza (2012). This will generate an additional term called the *interference term*. This term does not exist in classical probability and enables the representation of interferences between quantum states.

(10) Pr(b=β)=Classical_Probability(b=β)+Interference_Term

Under this representation, we can replace *Classical_Probability* by the classical law of total probability, and also replace the quantum *Interference_Term* by a measure of supplementary, represented by δ(β|*a, C*).

If we perform the normalization of the probability measure of supplementary δ(β|*a, C*) by the square root of the product of all probabilities, we obtain:

(11) $$\lambda_{\theta }=\frac{\delta (\beta |a,C)}{2\sqrt{\prod_{\alpha \in a}Pr(a=\alpha |C)Pr(b=\beta |a=\alpha ,C)}}$$

From Equation 11, the general probability formula of the Quantum-Like Approach can be derived. For two variables, is given by:

(12) $$\begin{array}{l} Pr(b=\beta |C)=\sum_{\alpha \in a}Pr(a=\alpha |C)Pr(b=\beta |a=\alpha ,C)\\ \;+2\lambda_{\theta }\sqrt{\prod_{\alpha \in a}Pr(a=\alpha |C)Pr(b=\beta |a=\alpha ,C)} \end{array}$$

If we look closely to Equation 12, we will see that the first summation of the formula corresponds to the classical law of total probability. The second term of the formula (the one that contains the λ_θ_ parameter), does not exist in the classical model and it is called the interference term.

In a quantum context, since the supplementary term δ(β|*a, C*) is being normalized in a quantum fashion, then we automatically know that the indicator term λ_θ_ will always have to be smaller than 1 in order to obtain quantum probabilities, λ_θ_ ≤ 1. So, under trigonometric contexts, the Quantum-Like Approach for quantum probabilities becomes:

(13) $$\begin{array}{l} \lambda_{\theta }=\cos(\theta )\;\;\;\to \;\;\;Pr(\beta |C)=\sum_{\alpha \in a}Pr(\alpha |C)Pr(\beta |\alpha ,C)\\ \;+2\sqrt{\prod_{\alpha \in a}Pr(\alpha |C)Pr(\beta |\alpha ,C)}\cos(\theta ) \end{array}$$

Equation 13 can be simplified in the following way:

(14) $$\begin{array}{l} Pr(\beta |C)=|\sqrt{Pr(\alpha_{1}|C)Pr(\beta |\alpha_{1},C)}\\ \;{+e^{i\theta_{\beta |\alpha ,C}}\sqrt{Pr(\alpha_{2}|C)Pr(\beta |\alpha_{2},C)}|}^{2} \end{array}$$

Equation 14 corresponds to the representation of the quantum law of total probability. In this equation, the angle θ_β|α, *C*_ corresponds to the phase of a random variable and incorporates the phase of both *A* = α_1_ and *A* = α_2_ in the following way: θ_β|α, *C*_ = θ_β|α1_ − θ_β|_α__2__.

One should note that, the Quantum-Like Approach can be extended to more complex decision scenarios, that is, with more than two random variables. However, this will lead to the very difficult task of tuning an exponential number of quantum θ parameters. Peter Nyman noticed this problem when he generalized the Quantum-Like Approach for three dichotomous variables (Nyman, 2010, 2011b; Nyman and Basieva, 2011a,b).

#### 4.2.1. The hyperbolic interference

Although the Quantum-Like Approach provides great possibilities comparing with the classical one, it seems that it cannot cover completely data from psychology and that a quantum formalism was not enough to explain some paradoxical findings (see Khrennikov et al., 2014), so hyperbolic spaces were proposed (Khrennikov, 2005c; Nyman, 2011a,b).

From Equation 12, if $Pr\left(b=\beta \right)-\underset{\alpha \in a}{\sum }Pr\left(a=\alpha |C\right)Pr\left(b=\beta |a=\alpha ,C\right)$ is different from zero, then some interference effects occur. In order to determine which type of interference happened, one tests the Quantum-Like Approach for quantum probabilities. This can be determined by normalizing the supplementary measure in a quantum fashion, just like presented in Equation 11.

If the probability *Pr*(*b* = β) was not computed in a trigonometric space (that is, it is not quantum), then, it is straightforward that the quantum normalization applied in Equation 11 will give a value bigger than 1. Since we are not in the context of quantum probabilities, the quantum normalization factor will fail to normalize the interference term, and will produce a number bigger than the normalization factor. Under these circumstances, the Quantum-Like Approach incorporates the generalization of *hyperbolic probabilities*, arguing that the context in which these probabilities were computed was in a Hyperbolic context (Khrennikov, 2009a, 2010; Nyman, 2011a).

Under Hyperbolic contexts, the Quantum-Like Approach contextual probability formula becomes:

(15) $$\begin{array}{l} \lambda_{\theta }=\cosh(\theta )\;\;\;\to \;\;\;Pr(\beta |C)=\sum_{\alpha \in a}Pr(\alpha |C)Pr(\beta |\alpha ,C)\\ \;\pm 2\sqrt{\prod_{\alpha \in a}Pr(\alpha |C)Pr(\beta |\alpha ,C)}\cosh(\theta ) \end{array}$$

In summary, according to the values computed by the indicator function λ_θ_, the Växjö Model enables the computation of probabilities in the following contexts:

- If |λ_θ_| = 0, then there is no interference and the Växjö Model collapses to classical probability theory.
- If |λ_θ_| ≤ 1, then we fall into the realm of quantum mechanics and the context becomes a Hilbert space. The indicator function is then replaced by the trigonometric function cos(θ).
- If |λ_θ_| > 1, then we fall into the realm of hyperbolic numbers and the context becomes a hyperbolic space. The indicator function is then replaced by the hyperbolic function cosh(θ).

### 4.3. The quantum prospect decision theory

The Quantum Prospect Decision Theory was developed by Yukalov and Sornette (2008, 2011) and developed throughout many other works (Yukalov and Sornette, 2009a,b, 2010a,b). The foundations of this theory are very similar to the previously presented Quantum-Like Approach.

In the Quantum-Like Approach, we start with two dichotomous observables. In the Quantum Prospect Decision Theory, these observables are referred to *intensions*. An intension can be defined by an intended action and a set of intended actions is defined by a *prospect*.

Each prospect can contain a set of action modes, which are concrete representations of an intension. Making a comparison with the Quantum-Like Approach, a prospect can be seen as a random variable and the set of action modes are the assignments that each random variable can have. For instance, the intension *to play* can have two representations: *play action A* or *play action B*.

Following the work of Yukalov and Sornette (2011), two intensions *A* and *B* with the respective representations: *A* = *x* where *x* ∈ *a*_1_, *a*_2_ and *B* = *y*, where *y* ∈ *b*_1_, *b*_2_. The corresponding state of mind is given by:

(16) $$|\;\psi_{s}\left(t\right)\rangle =\sum_{i,j}c_{i,j}\left(t\right)|\;A_{i}\;B_{j}\rangle$$

Equation 16 represents a linear combination of the prospect basis states. From a psychological perspective, the state of mind is a fixed vector characterizing a particular decision maker with his/her beliefs, habits, principles, etc. That is, it describes each decision maker as a unique subject.

The prospect states corresponding to the intensions *A* and *B* are given by Equation 17. The ψ symbol corresponds to quantum amplitudes associated with the prospect state. Under the Quantum Prospect Decision Theory, these amplitudes represent the weights of the intended actions, while a person is still deliberating about them.

(17) $$\begin{array}{l} |\pi_{A=a_{1}}\rangle =\psi_{11}|A=a_{1}B=b_{1}\rangle +\psi_{12}|A=a_{1}B=b_{2}\rangle \\ |\pi_{A=a_{2}}\rangle =\psi_{21}|A=a_{2}B=b_{1}\rangle +\psi_{22}|A=a_{2}B=b_{2}\rangle \end{array}$$

The probabilities of the prospects can be obtained by computing the squared magnitude of the prospect states (just like in the Quantum-Like Approach and the Quantum Dynamical Model). Consequently, the final probabilities are given by:

(18) $$\begin{array}{l} Pr(\pi_{A=a_{1}})=Pr(A=a_{1},B=b_{1})+Pr(A=a_{1},B=b_{2})\\ \;+Interference_{A=a_{1}}\\ Pr(\pi_{A=a_{2}})=Pr(A=a_{2},B=b_{1})+Pr(A=a_{2},B=b_{2})\\ \;+Interference_{A=a_{2}} \end{array}$$

Where the interference term in defined by:

(19) $$\begin{array}{l} Interference_{A=a_{1}}=2\cdot \varphi (\pi_{A=a_{1}})\sqrt{Pr(A=a_{1},B=b_{1})}\cdot \\ \;\sqrt{Pr(A=a_{1},B=b_{2})}\\ Interference_{A=a_{2}}=2\cdot \varphi (\pi_{A=a_{2}})\sqrt{Pr(A=a_{2},B=b_{1})}\cdot \\ \;\sqrt{Pr(A=a_{2},B=b_{2})} \end{array}$$

In Equation 19, the symbol φ corresponds to the uncertainty factor and is given by:

(20) $$\begin{array}{l} \varphi (\pi_{A=a_{1}})=\cos\left(arg\left(\psi_{11}\cdot \psi_{12}\right)\right)\\ \varphi (\pi_{A=a_{2}})=\cos\left(arg\left(\psi_{21}\cdot \psi_{22}\right)\right) \end{array}$$

The interference term corresponds to the effects that emerge during the process of deliberation, that is, while a person is making a decision. These interference effects result from conflicting interests, ambiguity, emotions, etc. (Yukalov and Sornette, 2011).

One can notice that the Quantum Prospect Decision Theory is very similar to the Quantum-Like Approach proposed by Khrennikov (2009c). Both theories end up with the same quantum probability formula. However, the Quantum Prospect Decision Theory provides some heuristics in how to choose the uncertainty factors. This information will be addressed in the next section.

#### 4.3.1. Choosing the uncertainty factor

In order to accommodate the violations of the Sure Thing Principle, the uncertainty factor must be set in such a way that it will enable accurate predictions. Two methods were proposed by Yukalov and Sornette (2011) to estimate the uncertainty factor: the Interference Alternation method and the Interference Quarter Law.

- **Interference Alternation**—Under normalized conditions, the probabilities of the prospects *p*(π_*j*_) must sum to 1. This normalization only occurs if one characterizes the interference term as an *alternation*, such that the interference effects disappear while summing the probability of the prospects. The interference alternation property is in accordance with the findings of Epstein (1999): the destructive interference effects can be associated with uncertainty aversion. This leads to a less probable action under uncertainty conditions. In contrast, the probabilities of other actions that contain less uncertainty are enhanced through constructive quantum interference effects. This uncertainty aversion happens quite frequently in situations where the Sure Thing Principle is violated. This implies that one of the probabilities of the prospects must be enhanced, whereas the other must be decreased.
  (21) $$\begin{array}{l} sign\left[\varphi (\pi_{A=a_{1}})\right]=-sign\left[\varphi (\pi_{A=a_{2}})\right]\;\;\;\;\;\;\text{where}\\ \;\;\left|\varphi (\pi_{A=a_{i}})\right|\in \left[0,1\right] \end{array}$$
- **Interference Quarter Law**—the interference terms generated by quantum probabilistic inferences, have a free quantum parameter, which is the uncertainty factor. The Interference Quarter Law corresponds to a quantitative estimation of this parameter. The modulus of the interference term *q* can be quantitatively estimated by computing the expectation value of the probability distribution of a random variable ξ in the interval [0, 1].
  (22) $$q\equiv \int_{0}^{1}\xi \cdot pr\left(\xi \right)\;d\xi =\frac{1}{4}$$
  The probability distribution *p*(ξ) is given by Equation 22 and can be computed by making the average of two probability distributions.
  (23) $$Pr\left(\xi \right)=\frac{1}{2}\left[pr_{1}\left(\xi \right)+pr_{2}\left(\xi \right)\right]=\delta \left(\xi \right)+\frac{1}{2}\Theta \left(1-\xi \right)$$

### 4.4. Quantum-Like Bayesian Networks in the literature

There are two main works in the literature that have contributed to the development and understanding of Quantum Bayesian Networks. One belongs to Tucci (1995) and the other to Leifer and Poulin (2008).

In the work of Tucci (1995), it is argued that any classical Bayesian Network can be extended to a quantum one by replacing real probabilities with quantum complex amplitudes. This means that the factorization should be performed in the same way as in a classical Bayesian Network.

One big problem with Tucci's work was the lack of methods to set the phase parameters. The author states that, one could have infinite Quantum Bayesian Networks representing the same classical Bayesian Network depending on the values that one chooses to set the parameter. This requires that one knows *a priori* which parameters would lead to the desired solution for each node queried in the network (which we never know). So, for these experiments, Tucci's model (Tucci, 1995) cannot predict the results observed, since one does not have any information about the quantum parameters.

In the work of Leifer and Poulin (2008), the authors argue that, in order to develop a quantum Bayesian Network, a quantum version is required of probability distributions, quantum marginal probabilities and quantum conditional probabilities (Table 3). The authors made a preliminary study on these concepts. Generally speaking, a quantum probability distribution corresponds to a density matrix contained in a Hilbert space, with the constraint that the trace of this matrix must sum to 1. In quantum probability theory, a full joint distribution is given by a *density matrix* ρ. This matrix provides the probability distribution of all states that a Bayesian Network can have. The marginalization operation corresponds to a quantum partial trace (Nielsen and Chuang, 2000; Rieffel and Polak, 2011). In the end, these models from the literature fail to provide any advantage relatively to the classical models, because they cannot take into account interference effects between random variables. So, they provide no advantages in modeling decision-making problems that try to predict decisions that violate the laws of total probability.

**Table 3:** **Relation between classical and quantum probabilities used in the work of Leifer and Poulin (2008)**.

	**Classical probability**	**Quantum probability**
State	*Pr*(*A*)	$|e^{i\theta }\psi_{A}{|}^{2}$
Joint probability distribution	*Pr*(*A, B*)	ρ_*AB*_
Marginal probability distribution	$Pr\left(B\right)=\underset{A}{\sum }Pr\left(A,B\right)$	ρ_*B*_ = *Tr*_*A*_(ρ_*AB*_)
Conditional state	*Pr*(*B*|*A*)	ρ_*B*|*A*_
	$\underset{b\in B}{\sum }Pr\left(b|A\right)$ = 1	*Tr*(ρ_*B*|*A*_) = *I*_*A*_

## 5. Problems with current classical and Quantum-Like approaches

In this section, we summarize the three main models that were presented in the previous sections (Table 4) and point out the advantages and disadvantages of each one of them.

**Table 4:** **Summary of the most relevant quantum decision models of the literature**.

**Model**	**State representation**	**Quantum interference**	**Predictive**	**Comments**
Quantum dynamical model	Superposition of subject's beliefs	Shröedinger's equation	No. requires manual fit	Enables time evolution
Quantum-Like approach	Contextual probabilities (observables/random variables)	Measure of supplementarity	No. requires manual fit	Can deal with hyperbolic spaces
Quantum prospect decision theory	Contextual probabilities (prospects/random variables)	Interference quarter law	Yes. Uses a static heuristic	It is predictive uses a heuristic
Quantum-Like Bayesian networks	Contextual probabilities (observables/random variables)	None	No. manual fit	Can easily scale to more complex scenarios

The Quantum-Like Approach is a very simple framework that enables the computation of quantum probabilities by performing the direct mapping between classical real probabilities and quantum probability amplitudes through Born's rule (Zurek, 2005, 2011). Although this model can be extended for *N* random variables and also go beyond quantum probabilities by incorporating hyperbolic spaces, this model cannot be called predictive, since there are no mechanisms to estimate the quantum θ parameters. One is required to know a priori the outcome of the decision scenario in order to fit the quantum parameters. So, this model has an explanatory nature in what concerns accommodating the paradoxical findings derived from violations of the Sure Thing Principle.

The Quantum Dynamical Model provides an elegant framework that can estimate decisions though time evolution. However, it also suffers from a major disadvantage related to Hamiltonian matrices. Creating a manual Hamiltonian is a very hard problem. It is required that all possible interactions of the decision problem are known and this specification must be made in such a way that the matrix is double stochastic. For more complex decision scenarios, this process is intractable. Furthermore, the Hamiltonian matrix grows exponentially with the complexity of the decision problem and the computation of a Unitary operator from such matrices is a very complex process. Most of the times, approximations are used, because of the complexity of the calculations involved in the matrix exponentiation operation.

The Quantum Prospect Decision Theory is a model very similar to the Quantum-Like Approach, but it is not extended to the hyperbolic spaces. The main advantage of the Quantum Prospect Decision Theory toward the other known quantum models is its predictive nature. The Quantum-Like Approach and the Quantum Dynamical model are more explanatory models. That is, they require that the outcome of an experiment is known in order to fit the parameters of the model and explain the paradoxical findings. The Quantum Prospect Decision Theory, on the other hand, contains an heuristic (the interference quarter law) that enables the estimation of the quantum parameters, turning the model predictive. However, the interference quarter law is a static heuristic. This means that, independently of the decision scenario and independently of the complexity of the decision, this interference term remains constant for every problem.

All of the above models exhibit different growth rates in parameters. For instance, the Dynamical Model parameterizes actions plus an additional parameter to model cognitive dissonance effects. So the number of parameters would be static if we consider the N-Person Prisoner's Dilemma Game. That is, instead of having only 2 players, this would be extended to N players. In the case of the Quantum-Like Approach, we would have 2^*N*^ parameters for the N-Person Prisoner's Dilemma Game. The number 2 comes from the fact that each player has two actions (either *Defect* or *Cooperate*). The same applies to the Quantum-Like Bayesian Networks and to the Quantum Prospect Theory Model. If we extend these models for *N* random variables, the number of parameters grows at a rate of $N_{actions}^{N_{person}}$, but these parameters will be automatically set using the Law of Quantum Interference, in the case of the Quantum Prospect Theory. The same is applied to the proposed Quantum-Like Bayesian Network, but instead of a static heuristic, we automatically set these parameters using a dynamic heuristic.

At this point, the reader might be thinking that the Quantum Dynamical Model provides great advantages toward the existing models, since the number of parameters required corresponds to the players actions with an additional cognitive dissonance parameter. Although this line of thought is correct, one should also take into account how the model unfolds. Although the numbers of parameters do not grow exponentially large as in the Quantum-Like Approach, the size of the Hamiltonian does. In fact, it grows exponentially large with the following size: $N_{actions}^{N_{players}}\times N_{actions}^{N_{players}}$, where *N*_*actions*_ represents the number of actions of the players and *N*_*players*_ corresponds to the number of players. The computation of a unitary operator from such matrices is a very complex process. Most of the times, approximations are used, because of the complexity of the calculations involved in the matrix exponentiation operation. Table 5 summarizes the parameter growth rate of each approach.

**Table 5:** **Comparison of the different growth rates in parameters for some models proposed in the literature**.

	**Approach**	**Parameter growth**	**Comments**
Busemeyer et al., 2009;	Quantum dynamical model	*N*_*actions*_	Hamiltonian size exponential:
Pothos and Busemeyer, 2009			$N_{actions}^{N_{person}}$
Khrennikov, 2009c	Quantum-Like approach	$N_{actions}^{N_{person}}$	Number of parameters
			Grows exponentially large
Yukalov and Sornette, 2011	Quantum prospect decision theory	$N_{actions}^{N_{person}}$	Static heuristic
			Interference quarter law

For these reasons, in this work, we propose a network structure framework that can easily scale to more complex decision scenarios. In other words, we propose a quantum-like Bayesian Network formalism, which consists in replacing classical probabilities by quantum probability amplitudes. However, since this approach also suffers from the problem of exponential growth of quantum parameters that need to be fit, we also propose a similarity heuristic that automatically computes this exponential number of quantum parameters (Shah and Oppenheimer, 2008).

## 6. A Quantum-Like Bayesian Network for decision and cognition

The reason why we chose Bayesian Networks is because it provides a link between probability theory and graph theory. And a fundamental property of graph theory is its modularity: one can build a complex system by combining smaller and simpler parts. It is easier for a person to combine pieces of evidence and to reason about them, instead of calculating all possible events and their respective beliefs (Griffiths et al., 2008). In the same way, Bayesian Networks represent the decision problem in small modules that can be combined to perform inferences. Only the probabilities, which are actually needed to perform the inferences, are computed.

**Figure 1:**
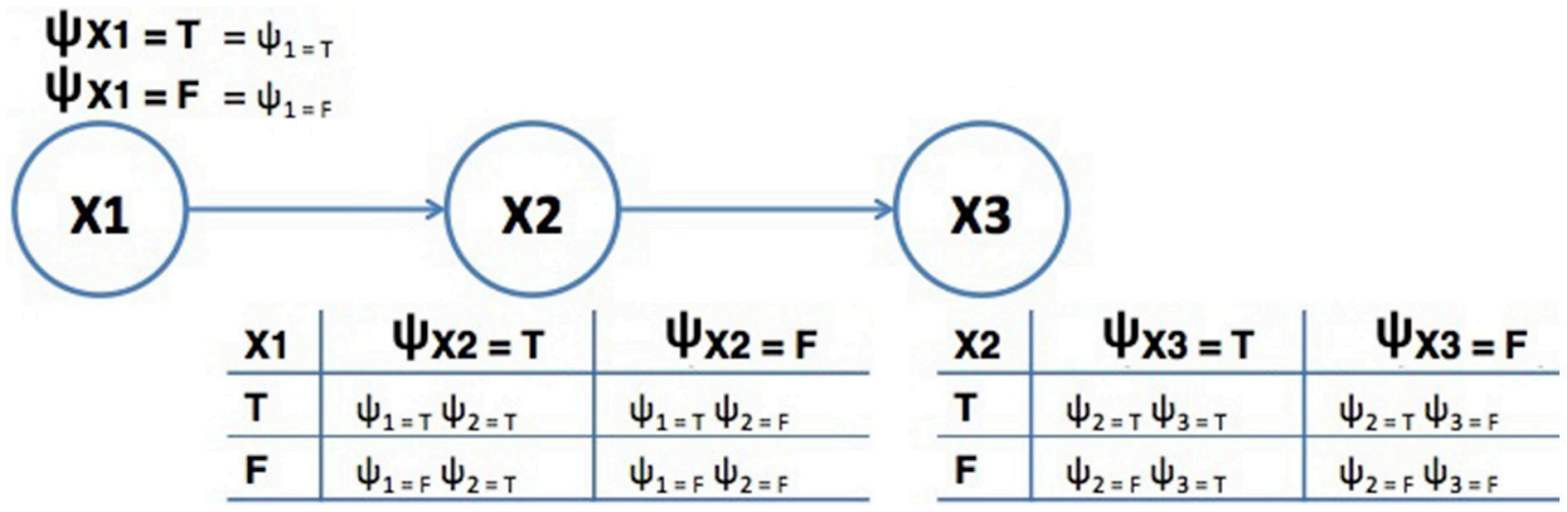
**Example of a Quantum-Like Bayesian Network**. The terms ψ_*i, j*_ correspond to quantum probability amplitudes. The variables *X*_1_, *X*_2_, and *X*_3_ correspond to random variables.

A Quantum-Like Bayesian Network can be defined in the same way as a classical Bayesian Network with the difference that real probability numbers are replaced by quantum probability amplitudes (Tucci, 1995). Figure 1 shows an example of the proposed Quantum-Like Bayesian Network, containing quantum probability amplitudes, ψ_*i, j*_, instead of real probability values.

In this sense, the quantum counterpart of the full joint probability distribution corresponds to the application of Born's rule to Equation 4. This results in the quantum like version of the full joint probability distribution:

(24) $$Pr(X_{1},\ldots ,X_{n})={\left|\prod_{i=1}^{n}\psi (X_{i}|Parents(X_{i}))\right|}^{2}$$

In order to perform exact inference in Bayesian Networks, the probability amplitude of each assignment of the network is propagated and influences the probabilities of the remaining nodes. That is, *every* assignment of *every* node of the Bayesian Network propagate throughout the network until they reach the node representing the query variable. Note that, by taking multiple assignments and paths at the same time, these trails influence each other producing interference effects.

The quantum counterpart of the Bayesian exact inference formula corresponds to the application of Born's rule to the classical marginal probability distribution equation (Equation 5).

(25) $$Pr(X|e)=\alpha {\left|\;\sum_{y}\prod_{x=1}^{N}\psi (X_{x}|Parents(X_{x}),e,y)\;\right|}^{2}$$

Expanding Equation 25, it will lead to the quantum marginalization formula with interference effects (Moreira and Wichert, 2014):

(26) $$Pr(X|e)=\alpha \sum_{i=1}^{\left|Y\right|}{\left|\prod_{x}^{N}\psi (X_{x}|Parents(X_{x}),e,y=i)\right|}^{2}+2\cdot Interference$$

$$\begin{array}{l} Interference=\\ \sum_{i=1}^{|Y|-1}\sum_{j=i+1}^{\left|Y\right|}\left|\prod_{x}^{N}\psi (X_{x}|Parents(X_{x}),e,y=i)\right|\cdot \\ \;\left|\prod_{x}^{N}\psi (X_{x}|Parents(X_{x}),e,y=j)\right|\cdot \cos(\theta_{i}-\theta_{j}) \end{array}$$

In the Quantum Dynamical Model, since it uses unitary operators, the double symmetric property of these operators does not require the normalization of the computed values. In the proposed approach, on the other hand, since we do not have the constraints of double stochastic operators, we need to normalize the final scores that are computed in order to achieve a probability value. In classical Bayesian inference, normalization of the inference scores is also necessary due to assumptions made in Bayes rule. The normalization factor corresponds to α in Equation 26.

Note that, in Equation 26, if one sets (θ_*i*_ − θ_*j*_) to π∕2, then cos(θ_*i*_ − θ_*j*_) = 0, which means that the quantum Bayesian Network collapses to its classical counterpart. That is, the proposed Quantum-Like Bayesian Network can behave in a classical way, if one sets the interference term to zero. Setting the angles to right angles means that all cosine similarities are 0 or 1. This transforms a continuous-valued system to a Boolean-valued system. Moreover, in Equation 26, if the Bayesian Network has *N* binary random variables, we will end up with 2^*N*^ free quantum θ parameters.

The proposed Bayesian Network leaves an open research question regarding the quantum θ parameters: *how can one compute such parameters in order to obtain realistic inferences?* By realistic, we mean the probability that an event that was observed in an experiment. These probabilities are impossible to compute using exact Bayesian inference in experiments where the Sure Thing Principle is being violated. In the next section, we answer this question by proposing a similarity heuristic that is able to compute the quantum θ parameters through vector similarities between beliefs/actions in superposition.

### 6.1. Representation of beliefs/actions

The superposition quantum vector, comprising all possible events, is given by the quantum full joint probability distribution already presented in Equation 24. The full joint probability distribution can be illustrated in table form just like it is presented in Table 6.

**Table 6:** **Table representation of a quantum full joint probability distribution**.

****X**_1_**	**…**	****X**_**N**_**	**ψ(**X**_*1*_, …, **X**_**N**_)**
T	⋯	T	$\psi_{1}\cdot e^{i\theta_{1}}$
T	⋯	F	$\psi_{2}\cdot e^{i\theta_{2}}$
⋮	⋮	⋮	⋮
F	…	F	$\psi_{M}\cdot e^{i\theta_{M}}$

The quantum probability inference formula is composed of two parts: one representing the classical probability and the other representing the quantum interference term. The interference term performs a summation over several combinations of the entries of the full joint probability distribution in groups of two variables: $\overset{N-1}{\underset{i=1}{\sum }}\overset{N}{\underset{j=i+1}{\sum }}|\psi_{i}||\psi_{j}|\cos\left(\theta_{i}-\theta_{j}\right)$. For each pair of variables, we will represent them as a 2-dimensional vector: one component represents the probability of ψ_*i*_ and the other corresponds to ψ_*j*_. Moreover, the different probabilities represented in the full joint probability distribution table can be seen as the different beliefs/actions that one might have available before making a decision.

(27) $$a(X=T)=\left[\begin{array}{c} {\left|\psi_{i}\cdot e^{i\theta_{i}}\right|}^{2}\\ {\left|\psi_{i}\cdot e^{i\theta_{j}}\right|}^{2} \end{array}\right]\;\;b(X=F)=\left[\begin{array}{c} {\left|\psi_{i}\cdot e^{i\theta_{i}}\right|}^{2}\\ {\left|\psi_{j}\cdot e^{i\theta_{j}}\right|}^{2} \end{array}\right]$$

**Figure 2:**
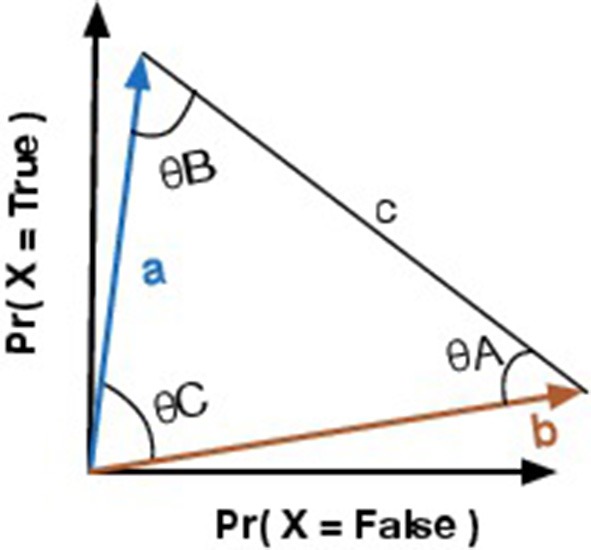
**Vector representation of two events representing a certain state**.

We always have two vectors, because the proposed Quantum-Like Bayesian network only supports binary random variables, that is, the query that it is performed to the network corresponds to a *yes* or *no* answer. In other words, one vector corresponds to the probability of the query random variable returning a positive answer, and the other corresponds to the probability of the query random variable returning a negative one. In a geometric space, these vectors are represented as in Figure 2. From these two vectors, similarity measures like the angles between the vectors or the distances between them can be computed. These similarity measures will be addressed in more detail in Section 6.2.

One could ask why these feature vectors are represented by probabilities. In our model, the goal is to find a quantum parameter that can be used to compute quantum probability inferences. The only information that one has are the probability distributions of a given scenario, which are encoded in the Bayesian Network.

In quantum mechanics, quantum states are always represented by unit length vectors. Since the proposed model is inspired by quantum formalisms, one might be wondering why the vectors are not unit length as well. There are two reasons for this choice. First, this representation of beliefs/actions as probabilities in feature vectors is not new, and it is a common practice in the literature (Osherson, 1995). Second, since our model is represented by a Bayesian Network and the vectors extracted directly from the network (through the representation of the full joint probability distribution), we do not need to have unit length vectors. Instead, this normalization will be performed during the inference process through the computation of the normalization factor α.

**Figure 3:**
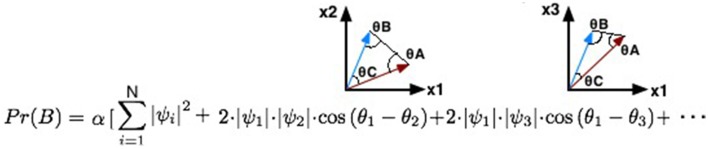
**Illustration of the different 2-dimensional vectors that will be generated for each step of iteration during the computation of the quantum interference term**.

In the end, the quantum interference term is computed by computing different vector representations for each pair of variables that are being computed (Figure 3). These vectors are extremely important to compute, since they will enable the calculation of different quantum θ parameters.

### 6.2. Acquisition of additional information

It is important to note that, over the current literature, quantum parameters must be assigned manually in order to obtain a prediction. So, for different experiments, we will have disparate quantum parameters. For this reason, it is very hard to create a universal heuristic that can assign quantum parameters for different applications. In this work, we propose a heuristic that is able to perform accurate predictions for the several different experiments reported in the literature related to the Prisoner's Dilemma Game and the Two Stage Gambling Game.

The goal of this similarity heuristic is to determine an angle between the vectors **a** and **b** (Equation 27) that can be used as the θ parameter in Equation 26. Moreover, by computing the Euclidean distance between vectors **a** and **b**, one can obtain vector **c**. Equation 28 shows how to obtain the norm of vector **c** through vectors **a** and **b** (Figure 2). Additional information is gained by comparing the similarity between the two vectors. This new information allows one to infer hidden properties of a participant's beliefs/actions from visible ones. This vector representation is similar to the approach proposed in the work of Pothos et al. (2013), where the authors represent a person's beliefs/actions in an *n*-dimensional vector space and the similarity between the vectors is measured by a projection operator, which corresponds to the computation of the squared length of the projected vector. This is similar to our approach, since we are also computing the length between the vectors **a** and **b**.

(28) $$\begin{array}{l} \left|\left|c\right|\right|=\\ \left|\left|a-b\right|\right|=\sqrt{{\left(a_{1}-b_{1}\right)}^{2}+{\left(a_{2}-b_{2}\right)}^{2}+\ldots +{\left(a_{n}-b_{n}\right)}^{2}} \end{array}$$

Since we are interested in the angles that these vectors make between each other, we used trigonometric laws, such as the law of cosines, to determine these angles. The law of cosines is given by Equations 29–31, where θ*A* corresponds to the angle between vectors **b** and **c**. θ*B* corresponds to the angle between vectors **a** and **c**. And θ*C* corresponds to the angle between vectors **a** and **b**. Since we know the coordinates of vectors **a** and **b**, one can also compute angle θ*C* through the similarity between two vectors using the cosine similarity measure: $\cos\left(\theta C\right)=\frac{\text{a}\cdot \text{b}}{\left||\text{a}||\cdot ||\text{b}|\right|}$. However, since we only know the length of vector **c**, we need to compare the similarity of the vectors through the law of cosines.

(29) $$\begin{array}{l} {\left|\left|a\right|\right|}^{2}={\left|\left|b\right|\right|}^{2}+{\left|\left|c\right|\right|}^{2}-2\cdot \left|\left|b\right|\right|\cdot \left|\left|c\right|\right|\cdot \cos\left(\theta A\right)\Leftrightarrow \theta A\\ \;=\cos^{-1}\left(\frac{{\left|\left|b\right|\right|}^{2}+{\left|\left|c\right|\right|}^{2}-{\left|\left|a\right|\right|}^{2}}{2\cdot \left|\left|b\right|\right|\cdot \left|\left|c\right|\right|}\right) \end{array}$$

(30) $$\begin{array}{l} {\left|\left|b\right|\right|}^{2}={\left|\left|a\right|\right|}^{2}+{\left|\left|c\right|\right|}^{2}-2\cdot \left|\left|a\right|\right|\cdot \left|\left|c\right|\right|\cdot \cos\left(\theta B\right)\Leftrightarrow \theta B\\ \;=\cos^{-1}\left(\frac{{\left|\left|a\right|\right|}^{2}+{\left|\left|c\right|\right|}^{2}-{\left|\left|b\right|\right|}^{2}}{2\cdot \left|\left|a\right|\right|\cdot \left|\left|c\right|\right|}\right) \end{array}$$

(31) $$\begin{array}{l} {\left|\left|c\right|\right|}^{2}={\left|\left|a\right|\right|}^{2}+{\left|\left|b\right|\right|}^{2}-2\cdot \left|\left|a\right|\right|\cdot \left|\left|b\right|\right|\cdot \cos\left(\theta C\right)\Leftrightarrow \theta C\\ \;=\cos^{-1}\left(\frac{{\left|\left|a\right|\right|}^{2}+{\left|\left|b\right|\right|}^{2}-{\left|\left|c\right|\right|}^{2}}{2\cdot \left|\left|a\right|\right|\cdot \left|\left|b\right|\right|}\right) \end{array}$$

### 6.3. Definition of the similarity heuristic

Violations to the Sure Thing principle imply a decrease in the final probability values when compared to the classical theory. This suggests that, somehow, we need to force the quantum parameters to have a destructive interference effect. This can be obtained by setting the quantum parameter to π (which is the angle that provides the smallest cosine value). The additional information that we incorporated in Figure 2, namely the Euclidean distance between vectors and their similarities, is translated into a triangle. This shape has a well-known property that all their inner angles must sum to 180° or π radians. Moreover, we would like to have a destructive interference effect that takes into account the similarity of the original vectors. Equation 32, shows how one can obtain this relationship.

(32) $$\begin{array}{l} \theta_{A}+\theta_{B}+\theta_{C}=\pi \Leftrightarrow \pi -\theta_{C}=\theta_{A}+\theta_{B}\\ \;\;\Leftrightarrow \pi -\frac{\theta_{C}}{2}=\frac{\theta_{A}+\theta_{B}+\pi }{2} \end{array}$$

When, the similarity of the vectors is very small, that is θ_*C*_ is very small, then we can add a third relationship:

$$\theta_{A}+\theta_{B}+\theta_{C}=\pi \Leftrightarrow \pi =\theta_{A}+\theta_{B}$$

In this sense, we can formulate the general formula of the proposed similarity heuristic :

(33) $$h\left(\;a,\;b\;\right)=\{\begin{array}{l} \pi \;\;\;\;\;\;\;\;\;\;\;\;\;\;\;\;\;\;\;\text{if}\phi <\text{0}\\ \pi -\theta_{C}/2\;\;\;\;\;\;\;\text{if}\phi >\text{0}.\text{2}\\ \pi -\theta_{C}\;\;\;\;\;\;\text{otherwise} \end{array}$$

We also came up with a similarity measure ϕ, which is given by the ratio between all the angles that the vectors make between them. In order words, it represents the similarity between the additional information found by manipulating the original vectors and is given by Equation 34.

(34) $$\phi =\frac{\theta_{C}}{\theta_{A}}-\frac{\theta_{B}}{\theta_{A}}$$

The thresholds shown in the proposed similarity heuristic were taken by observing the data from several experiments violating the Sure Thing Principle. These include several experiments in the literature of the Prisoner's Dilemma Game and the Two Stage Gambling Game. Yukalov and Sornette (2011) also did something similar. They analyzed the experiments violating the Sure Thing Principle and came up with a static interference term (the Interference Quarter Law) that allows them to apply their model without knowing exactly a priori the outcome of some specific experiment. The proposed model works under similar conditions. We analyzed several experiments from the literature from different games and mapped the trends of the data into a dynamic heuristic. So, in the end, the proposed model works under some rules that enables a dynamic behavior (after all each experiment is unique, so there should be the freedom of different quantum interferences) and also enables the application of the model without specific a priori knowledge from a specific experiment.

In quantum mechanics, the θ parameter corresponds to the phase of a wave. When representing a quantum state in a Hilbert space, this phase is given by the inner product between two quantum states (Busemeyer and Bruza, 2012). The proposed similarity heuristic is motivated by the same idea. For two vectors representing a person's belief/action, we find which angle (or in this case, a combination of angles) that can lead to the observed probabilities for the Prisoner's Dilemma and for the Two Stage Gambling game.

### 6.4. Summary of the proposed model

The proposed model is built based on observed data to perform quantum probabilistic inferences. We are using a similarity heuristic, which relies in the data of the Bayesian Network to indicate the parameters that will allow us to perform quantum probabilistic inferences. One should keep in mind that this function is a heuristic: it generally provides good results in many situations (in this case, the Two Stage Gambling game, and the Prisoner's Dilemma), but at the cost of occasionally not giving us very accurate results (Shah and Oppenheimer, 2008).

In sum, the proposed model works as follows:

- Definition of a quantum-like Bayesian Network containing cause/effect relationships of a given scenario. Each node of the Bayesian Network corresponds to a binary random variable and is associated to a conditional probability table. These tables represent conditional probability distributions, which can be converted to quantum amplitudes through Born's rule.
- When performing a query to the quantum-like Bayesian Network, a set of quantum parameters will emerge, because of the application of Equation 26. These parameters can be determined with the similarity heuristic that takes into account similarities between vectors.
- The proposed similarity heuristic takes into account two 2-dimensional vectors. Each vector corresponds to one assignment of the query variable (for instance, the probability of the query being true or the probability of the query being false).
- The two features of each vector correspond to each entry of the full joint probability distribution of the Bayesian Network that has the same assignment of the query variable. For instance, all entries of the distribution that have the assignment of the query variable set to *true*.
- After knowing the similarities that the vectors share between them, we can apply the proposed similarity heuristic given in Equation 33 to obtain a θ parameter that enables the computation of the final probability value of the query.

One might be thinking that we use two of the three data points directly in the model (known *Defect* and known *Collaborate*). Then, they use one free parameter to account for the remaining data point (the probability of Defection in the unknown condition). However, this is not what we state with this work. As already mentioned, this work is a nonparametric method for estimating inference effects from a statistical point of view. It is a statistical model that is simpler than the previous quantum dynamic and quantum-like models proposed in the literature. Again, this work is not about simulation methods of fitting. We are simply providing a Bayesian Network structure that enables a simple representation of more complex decision-making scenarios, and the incorporation of a similarity heuristic (which results from algebraic manipulations) in order to assign values to quantum parameters in such a way that provides accurate predictions (that is, it can represent the data accurately).

In the next sections, we will present a full example of how the proposed Quantum-Like Bayesian Network can be applied (Section 6.5). We will also present experimental results of the proposed model applied to several works of the literature concerned with the Prisoner's Dilemma game (Section 7.1) and the Two Stage Gambling game (Section 7.2).

### 6.5. Example of application of the proposed model

**Figure 4:**
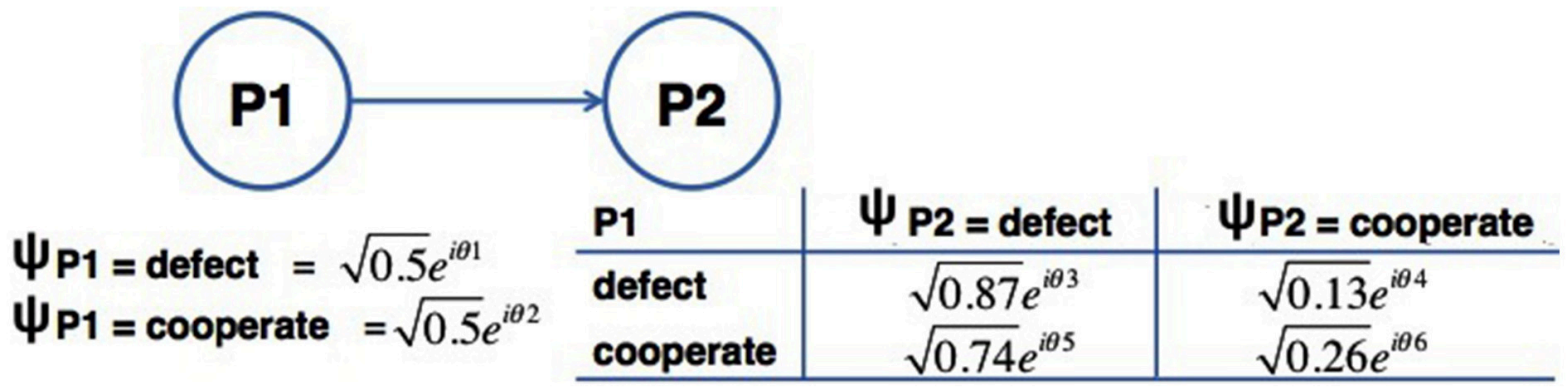
**Bayesian Network representation of the Average of the results reported in the literature (last row of Table 1)**. The random variables, which were considered, are P1 and P2, corresponding to the actions chosen by the first participant and second participant, respectively.

**Figure 5:**
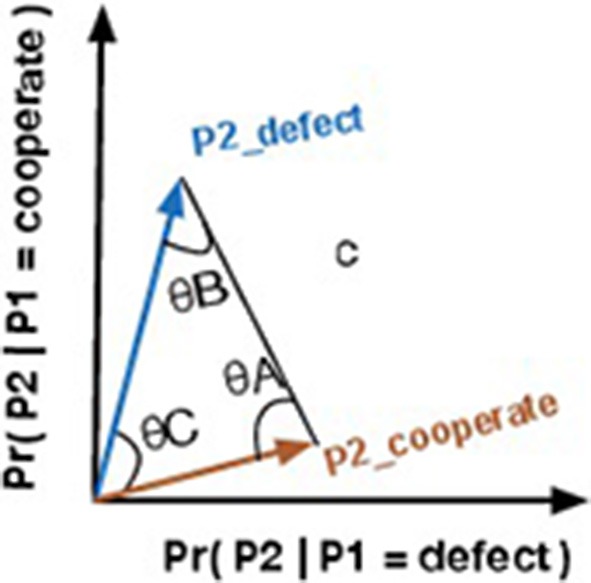
**Vector representation of events P2_*Defect*_ and P2_*Cooperate*_ plus the euclidean distance vector *c***.

In this section, we will demonstrate how the proposed Bayesian Network can be applied to the average results presented in Table 1 for the Prisoner's Dilemma game. The proposed Quantum-Like Bayesian Network can be summarized in the following steps:

- **Step 1: Create a Bayesian Network Representation of the Problem:** In the Prisoner's dilemma game, if nothing is told to the participants, then there is a 50% chance of the first participant choosing to *Defect* or *Cooperate*. The decision of the first participant is then followed by the decision of the second participant. A Bayesian Network representation of this problem is illustrated in Figure 4.
- **Step 2: Compute the Vectors associated to each action**. Since we want to determine the *Pr*(*P*2 = *Defect*), this probability will be given by the quantum full joint probability distribution, which is represented in Table 7.
  (35) $$\begin{array}{l} \;P2_{Defect}=\left[\begin{array}{c} {\left|0.6595\cdot e^{i\cdot \theta_{A}}\right|}^{2}\\ {\left|0.6083\cdot e^{i\cdot \theta_{C}}\right|}^{2} \end{array}\right]=\left[\begin{array}{c} 0.435\\ 0.370 \end{array}\right]\\ P2_{Cooperate}=\left[\begin{array}{c} {\left|0.2550\cdot e^{i\cdot \theta_{B}}\right|}^{2}\\ {\left|0.3606\cdot e^{i\cdot \theta_{D}}\right|}^{2} \end{array}\right]=\left[\begin{array}{c} 0.065\\ 0.130 \end{array}\right] \end{array}$$
  This way, one can build feature vectors using classical probabilities. For instance, the probability of *Pr*(*P*2 = *Defect*) is given by a 2-dimensional feature vector with entries: *Pr*(*P*1 = *Defect*) · *Pr*(*P*2 = *Defect*|*P*1 = *Defect*) and *Pr*(*P*1 = *Cooperate*) · *Pr*(*P*2 = *Defect*|*P*1 = *Cooperate*). The feature vector corresponding to the action *Cooperate* can be achieved in the same way (Equation 35).
- **Step 3: Determine the quantum parameters using the proposed similarity heuristic:** Since we only have two random variables, we only need to compute one θ parameter. This parameter can be obtained by directly by first computing the Euclidean distance between **P2**_*Defect*_ and **P2**_*Cooperate*_, and by computing the inner angles of the resulting triangle (Figure 5).
  (36) $$\begin{array}{l} \left|\left|c\right|\right|=\left|\left|P2_{Defect}-P2_{Cooperate}\right|\right|\\ \;=\sqrt{{\left(0.435-0.065\right)}^{2}+{\left(0.37-0.13\right)}^{2}}=0.4410 \end{array}$$
  The norm of vectors *P*2_*Defect*_ and *P*2_*Cooperate*_ is given by:
  (37) $$\begin{array}{l} \left|\left|P2_{Defect}\right|\right|=\sqrt{0.435^{2}+0.370^{2}}=0.5711\\ \left|\left|P2_{Cooperate}\right|\right|=\sqrt{0.065^{2}+0.130^{2}}=0.1453 \end{array}$$
  The inner angles of the triangle formed by vectors **P2**_*Defect*_ and **P2**_*Cooperate*_ and **c** can be computed from the law of Cosines presented in Equations 38–40.
  (38) $$A=\cos^{-1}\left(\frac{{\left|\left|P2_{Cooperate}\right|\right|}^{2}-{\left|\left|P2_{Defect}\right|\right|}^{2}+c^{2}}{2\cdot c\cdot \left|\left|P2_{Cooperate}\right|\right|}\right)=2.6102$$
  (39) $$B=\cos^{-1}\left(\frac{{\left|\left|P2_{Defect}\right|\right|}^{2}-{\left|\left|P2_{Cooperate}\right|\right|}^{2}+c^{2}}{2\cdot c\cdot \left|\left|P2_{Defect}\right|\right|}\right)=0.1294$$
  (40) $$C=\cos^{-1}\left(\frac{{\left|\left|P2_{Defect}\right|\right|}^{2}+{\left|\left|P2_{Cooperate}\right|\right|}^{2}-c^{2}}{2\cdot \left|\left|P2_{Defect}\right|\right|\cdot \left|\left|P2_{Cooperate}\right|\right|}\right)=0.4023$$
  Given that $\frac{\theta C}{\theta A}-\frac{\theta B}{\theta A}=0.1046$, then the final quantum θ parameter can be computed by using the third condition of Equation 33
  (41) θ=π − θC =π−0.4023=2.7393
- **Step 4: Perform the Probabilistic Inference**. In order to compute *Pr*(*P*2 = *Defect*) we also need to compute the opposite probability, that is, *Pr*(*P*2 = *Cooperate*). Equation 42 represents quantum amplitudes through the symbol ψ. The sub indexes *D* and *C* correspond to the actions *Defect* and *Cooperate*, respectively.
  (42) $$\begin{array}{l} Pr(P2=Defect)=\alpha [{\left|\psi_{P2=D|P1=D}\right|}^{2}+{\left|\psi_{P2=D|P1=D}\right|}^{2}\\ \;+2\cdot \left|\psi_{P2=D|P1=D}\right|\cdot \left|\psi_{P2=D|P1=C}\right|\cdot \cos\left(\theta \right)] \end{array}$$
  (43) $$\begin{array}{l} Pr(P2=Defect)=\alpha [0.5\times 0.87+0.5\times 0.74\\ \;+2\times \sqrt{0.5\times 0.87}\times \sqrt{0.5\times 0.74}\cos\left(2.7393\right)] \end{array}$$
  Computing the probability of *Pr*(*P*2 = *Cooperate*) in the same way, we obtain:
  (44) $$\begin{array}{l} Pr(P2=Defect)=\alpha \cdot 0.0667\\ Pr(Cooperate)=\alpha \cdot 0.0258 \end{array}$$
- Step 5: Compute Normalization Factor and Final Probabilities.
  (45) $$\alpha =\frac{1}{0.0667+0.0258}=\frac{1}{0.0925}=10.8108$$
  The final probabilities are given by Equation 45. Note that in Table 1, the observed probability of a player choosing to *Defect* was 0.64. The proposed Bayesian Network estimated this probability to be approximately 0.72, which corresponds to a fit error percentage of 12.63%.
  (46) Pr(P2=Defect)=0.7208     Pr(P2=Cooperate)=0.2792

**Table 7:** **Quantum full joint probability distribution representation of the Bayesian Network in Figure 4**.

**P1**	**P2**	**Pr(P1, P2)**
*Defect*	*Defect*	$\sqrt{0.5}\cdot e^{i\cdot \theta_{1}}\times \sqrt{0.87}\cdot e^{i\cdot \theta_{3}}=0.6595\cdot e^{i\cdot \left(\theta_{1}+\theta_{3}\right)}$
		$\;=0.6595\cdot e^{i\cdot \theta_{A}}$
*Defect*	*Cooperate*	$\sqrt{0.5}\cdot e^{i\cdot \theta_{1}}\times \sqrt{0.13}\cdot e^{i\cdot \theta_{4}}=0.2550\cdot e^{i\cdot \left(\theta_{1}+\theta_{4}\right)}$
		$\;=0.2550\cdot e^{i\cdot \theta_{B}}$
*Cooperate*	*Defect*	$\sqrt{0.5}\cdot e^{i\cdot \theta_{2}}\times \sqrt{0.74}\cdot e^{i\cdot \theta_{5}}=0.6083\cdot e^{i\cdot \left(\theta_{2}+\theta_{5}\right)}$
		$\;=0.6083\cdot e^{i\cdot \theta_{C}}$
*Cooperate*	*Cooperate*	$\sqrt{0.5}\cdot e^{i\cdot \theta_{2}}\times \sqrt{0.26}\cdot e^{i\cdot \theta_{6}}=0.3606\cdot e^{i\cdot \left(\theta_{2}+\theta_{6}\right)}$
		$\;=0.3606\cdot e^{i\cdot \theta_{D}}$

## 7. Experimental results

Violations to the Sure Thing Principle are hard to verify in complex decision-making problems. For this reason, there is not much data available in the literature for validation purposes. So, in this work, we will validate our model for several different experiments made to detect violations of the Sure Thing Principle in the Prisoner's Dilemma Game (Section 7.1) and for the Two Stage Gambling game (Section 7.2).

### 7.1. Quantum Bayesian Network applied to the Prisoner's Dilemma game

In this section, we apply our model to predict the results obtained for the Prisoner's Dilemma game for several works in the literature.

It is common (and good) practice in cognitive science to compare the results of one's model to the results of leading comparable models. The fit error percentages that we present in the following sections would be much easier to interpret if there could be other models to compare with. However, we cannot perform this comparison directly, because the current models of the literature only work for isolated experiments, just like it was shown for the Quantum Dynamical Model (Section 4.1) and the Quantum-Like Approach (Section 4.2). That is, each time there is a new experiment, the parameters of their respective models would need to be tuned manually in order to perform correct predictions. We propose a general and scalable framework that is able to perform predictions in several different setting with small amounts of fit errors.

**Figure 6:**
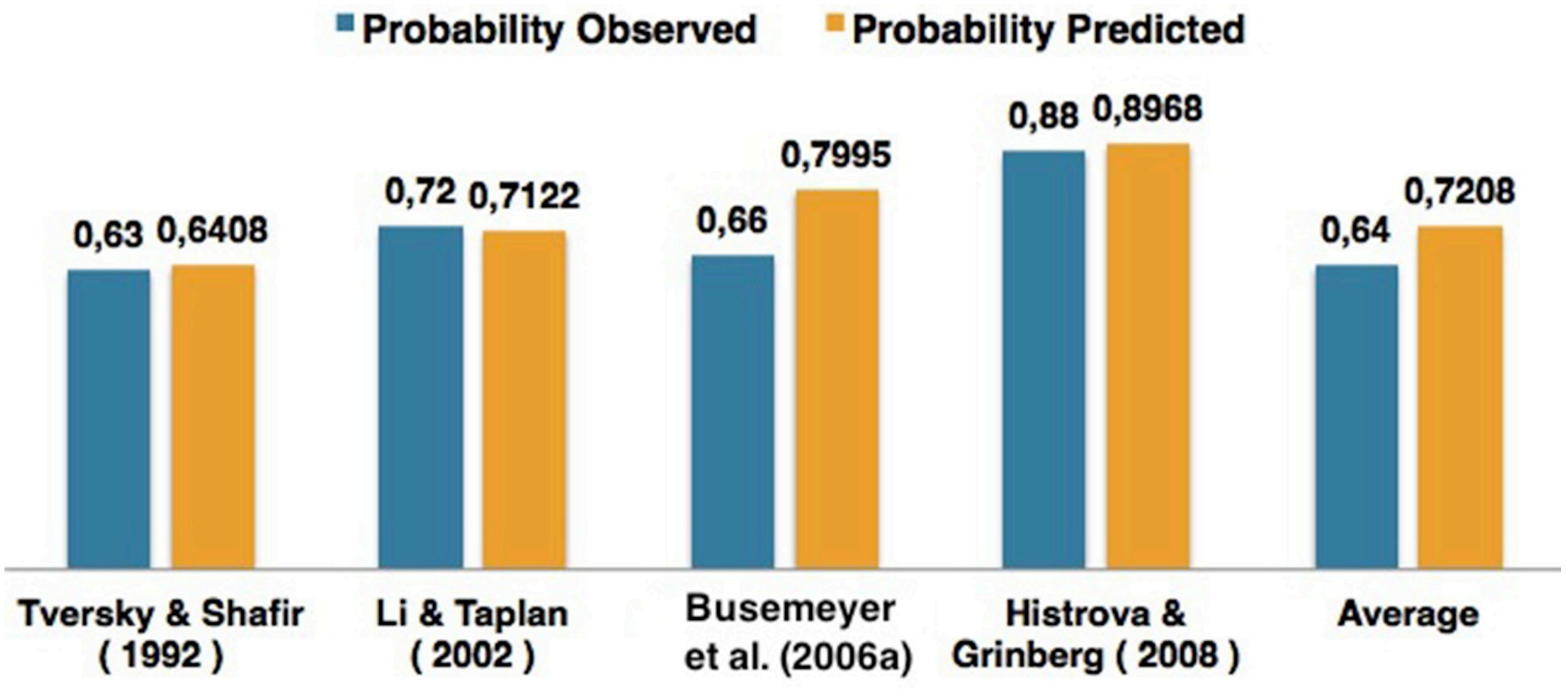
**Comparison of the results obtained for different works of the literature concerned with the Prisoner's Dilemma game**.

In this sense, we modeled each result reported in Table 1 with the proposed Bayesian Network and using the proposed similarity heuristic. We obtained the results that are presented in Figure 6.

For a more detailed analysis of Figure 6, Table 8 shows the quantum θ parameters that were computed for each experiment and the quantum parameter that would be expected to achieve a 0% fit error. The fit error is a percentage value and was computing in the following way: $\left(1-\frac{computed\text{\_}probability}{observed\text{\_}probability}\right)*100$. In Table 8, the term *computedprobability* corresponds to the column *Pr(Defect) predicted* and the term *observed_probability* corresponds to the column *observed_probability*.

**Table 8:** **Analysis of the quantum θ parameters computed for each work of the literature using the proposed similarity function**.

**Literature**	**Expected θ**	**Computed θ**	**Pr(Defect)**	**Pr(Defect) predicted**	**Fit error**
Shafir and Tversky, 1992	2.8151	2.8057	0.6300	0.6408	1.71
Li and Taplin, 2002^b^	3.3033	3.0121	0.7200	0.7122	1.09
Busemeyer et al., 2006a	2.9738	3.3628	0.6600	0.7995	21.13
Hristova and Grinberg, 2008	2.8255	2.7400	0.8800	0.8968	3.01
Average	2.8718	2.7393	0.6400	0.7208	12.63

*Expected θ corresponds to the quantum parameter that leads to the observed probability value in the experiment. Computed θ corresponds to the quantum parameter computed with the proposed heuristic*.

b *corresponds to the average of all seven experiments reported*.

In Table 8, one can see that the proposed similarity heuristic was able to perform good approximations to the data. The dynamical heuristic enabled to perform different estimations of quantum interference effects for different decision problems. However, since it is an heuristic, it can sometimes lead to overestimations, which was the case in the work of Busemeyer et al. (2006a). These overestimations occur due to the sensitivity of the quantum parameters. That is, a small change in a quantum parameter will lead to a completely different probability value. This will be discussed in more depth in Section 7.1.2.

As one might have noticed, the work of Croson (1999) was not taken into account in the analysis of these results. We decided to analyse these results in the next section, because they contained properties that were different from the remaining works. In Croson (1999), the participants were never told about the actions of the other player. The author asked for the participants to first try to guess what action the other player chose and then make a decision. In another setting, participants were just asked to make a decision.

#### 7.1.1. The special case of Croson's (1999) experiments

In work of Croson (1999), we used the results reported for the first two payoff matrices tested in their work and performed the average of the results. When trying to compute the optimum quantum θ parameter that would lead to the computation of the probability with a 0% fit error, we could not find any. There was no possible parameter that could be obtained from the two feature vectors representing the probability of choosing either a *Defect* action or a *Cooperate* action.

As a first thought, we noticed that the average of the results could be the cause of such impossibility, because they were not the true probabilities of the events reported. So, we decided to analyse the outcome of each experiment of the work of Croson (1999) individually. Table 9 specifies those results.

**Table 9:** **Results for the two games reported in the work of Croson (1999) for the Prisoner's Dilemma Game for several conditions: when the action of the second player was guessed to be *Defect* (Guessed to Defect), when the action of the second player was guessed to be *Cooperate* (Guessed to Collaborate), and when the action of the second player was not known (Unknown)**.

**Croson, 1999**	**Guessed to Defect**	**Guessed to Cooperate**	**Unknown**	**Unknown predicted**	**Violation of STP**
Game 1	0.1700	0.6800	0.2250	0.5877	No
Game 2	0.4700	0.6500	0.3750	0.4390	Yes
Average	0.6700	0.32	0.3000	0.5053	Yes

**Figure 7:**
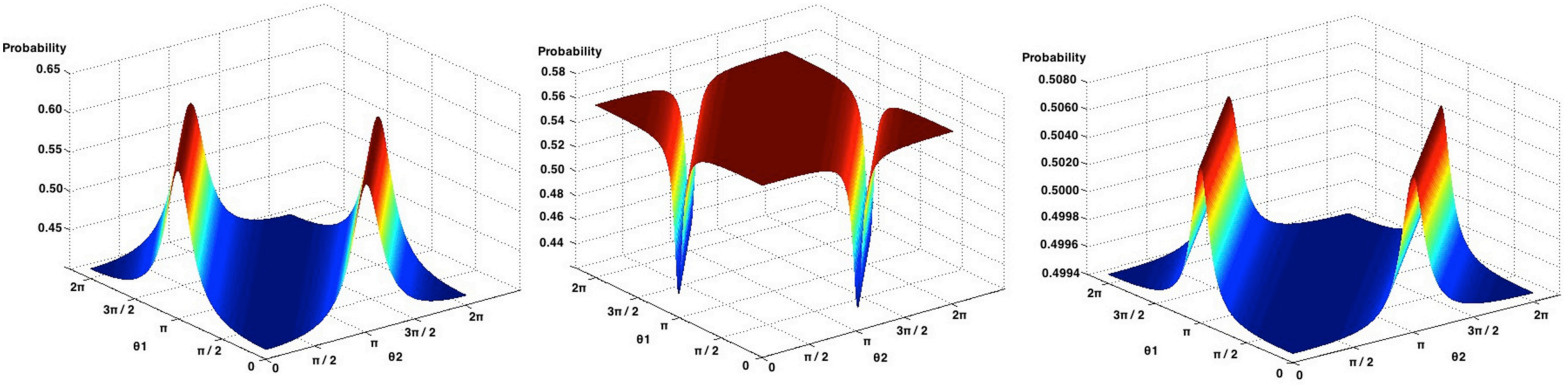
**Possible probabilities that can be obtained from Game 1 (left), Game 2 (center) and the average of the Games of the work of Croson (1999), using the quantum law of total probability**.

We again analyzed the individual results of Table 9, and again, we could not find any quantum θ parameter that would lead to the computation of probabilities with a 0%. On the contrary, the minimum fit errors found were 64.89, 83.25, and 17.06% for Game 1, Game 2 and the Average of these games, respectively. Figure 7 present all possible probabilities that can be computed using the quantum law of total amplitude.

Analysing Game 1 (Figure 7, left), the probability that leads to the smallest fit error is obtained when both θ parameters are set to zero, with a probability of 0.4123. The observed probability reported in this experiment corresponds to 0.2250, leading to a computed fit error of 64.69%.

For Game 2 (Figure 7, center), when θ_1_ = 0 and θ_2_ = π, we obtain the probability that leads to the smallest fit error, which is 0.4390, with a fit error of 83.25 %.

When computing the average of both games (Figure 7, right), the quantum θ parameters found were θ_1_ = 0 and θ_2_ = 0. This leads to a probability of 0.4947, corresponding to a fit error of 17.06%.

#### 7.1.2. Analysing Li and Taplin (2002) experiments

Table 10 specifies the results collected by Li and Taplin (2002), which corresponded to the average of the results obtained in seven different experiments for the Prisoner's Dilemma game. In this section we analyse each of these seven experiments, by trying to predict their outcome using the proposed Bayesian Network.

**Table 10:** **Experimental results reported in work of Li and Taplin (2002) for the Prisoner's Dilemma game for several conditions: when the action of the second player is known to be *Defect* (Known to Defect), when the action of the second player is known to be *Cooperate* (Known to Collaborate), and when the action of the second player was not known (Unknown)**.

**Li and Taplin, 2002**	**Known Defect**	**Known Cooperate**	**Unknown**	**Classical probability**	**Violation of STP**
Game 1	0.7333	0.6670	0.6000	0.7000	Yes
Game 2	0.8000	0.7667	0.6300	0.7833	Yes
Game 3	**0.9000**	**0.8667**	**0.8667**	**0.8834**	**No**
Game 4	0.8333	0.8000	0.7000	0.8167	Yes
Game 5	0.8333	0.7333	0.7000	0.7833	Yes
Game 6	**0.7667**	**0.8333**	**0.8000**	**0.8000**	**No**
Game 7	**0.8667**	**0.7333**	**0.7667**	**0.8000**	**No**
Average	0.8200	0.7700	0.7200	0.7950	Yes

*The column Violations of STP corresponds to determining if the collected results are violating the Sure Thing Principle. The values in bold represent the experiments that are not violating the Sure Thing Principle*.

The results reported in the experiments conducted by Li and Taplin (2002) are presented in Table 10. Note that Games 3, 6 and 7 are not violating the Sure Thing Principle, because: *Pr* (*Defect*) ≥ *Pr* (*Unknown*) ≤ *Pr* (*Cooperate*) or *Pr* (*Cooperate*) ≥ *Pr* (*Unknown*) ≤ *Pr* (*Defect*). Additionally, the results reported for the unknown condition in Games 3, 6 and 7 are very close to the classical probability theory. The goal of the study performed by Li and Taplin was to question if there was really violations of the Sure Thing Principle under the Prisoner's Dilemma game. According to Table 10 three of the seven experiments did not show a violation, and reported results very similar to the classical probability theory.

**Figure 8:**
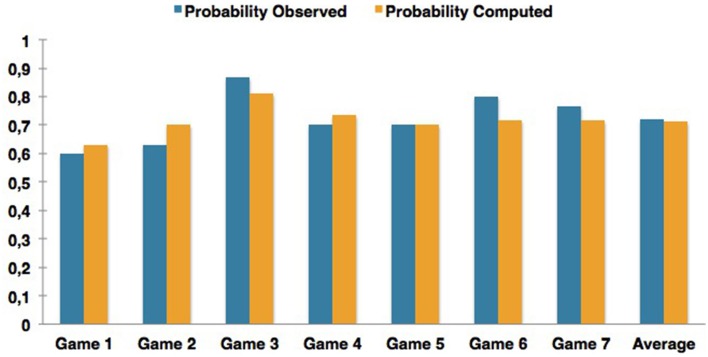
**Comparison of the results obtained for different experiments reported in the work of Li and Taplin (2002) in the context of the Prisoner's Dilemma game**.

By applying the proposed quantum-like Bayesian Network each game in Table 10, we obtained the results illustrated in Figure 8.

The experiments that achieved the highest fit error rates correspond to Games 2 and 6. Game 6 corresponds to a situation where the Sure Thing Principle was not being violated. This leads to the conclusion that the proposed Bayesian Network can also predict classical probabilities, but with some fit errors.

**Figure 9:**
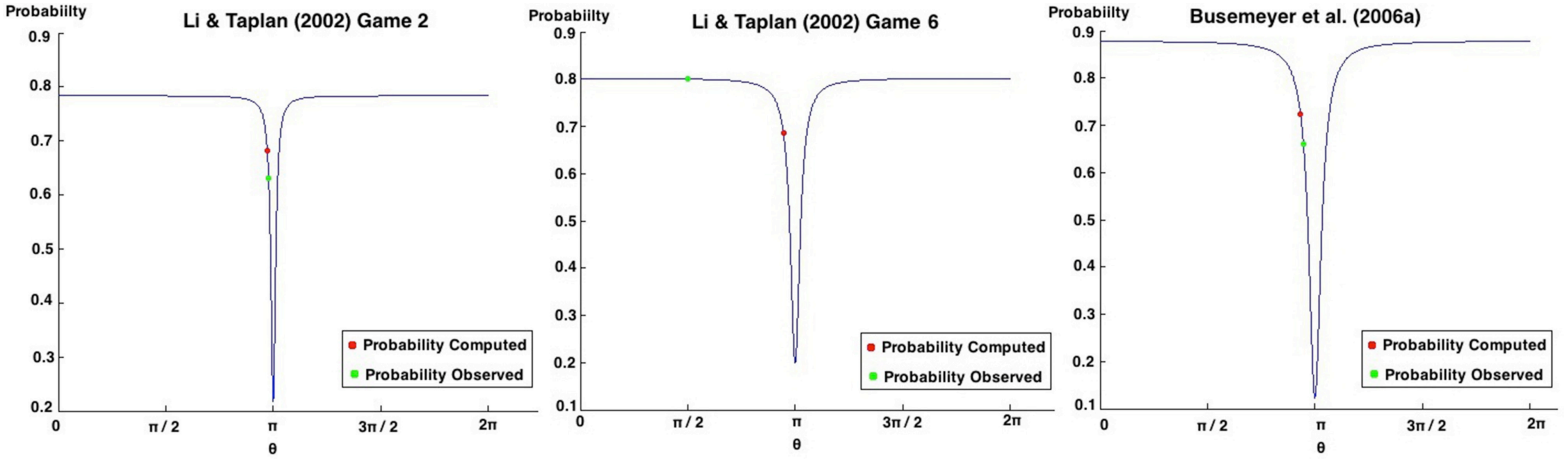
**Possible probabilities that can be obtained in Game 2 of the work of Li and Taplin (2002) (left)**. Possible probabilities that can be obtained in Game 6 of the work of Li and Taplin (2002) (center). Possible probabilities that can be obtained in the work of Busemeyer et al. (2006a) (right).

Table 11 shows the quantum parameters that were computed and compares them with the parameters that would be expected in order to obtain the smallest fit error percentage. One thing worth mentioning in the computation of these quantum parameters is their sensitivity. Consider the row of Table 11 addressing the results of Game 2. The difference between expected quantum parameter with the one that was computed using the similarity heuristic corresponds to a difference of just 0.0322. However, this small difference introduced a fit error of almost 11.28% in the computation of the final probabilities. Figure 9 illustrates the relation between the quantum θ parameter and the final probabilities that can be obtained in Li's Game 2, Game 6 and the work of Busemeyer et al. (2006a).

**Table 11:** **Experimental results reported in work of Li and Taplin (2002) for the Prisoner's Dilemma game**.

**Li and Taplin, 2002**	**Expected θ**	**Computed θ**	**Unknown**	**Unknown predicted**	**Fit error %**
Game 1	3.0170	2.9845	0.6000	0.6313	5.21
Game 2	3.0758	3.0436	0.6300	0.7011	11.28
Game 3	**2.8052**	**2.9810**	**0.8667**	**0.8113**	**6.39**
Game 4	3.2313	3.0306	0.7000	0.7341	4.87
Game 5	2.8519	2.8511	0.7000	0.7006	0.08
Game 6	**1.5708**	**2.9350**	**0.8000**	**0.7169**	**10.39**
Game 7	**3.7812**	**2.7365**	**0.7667**	**0.7159**	**6.63**
Average	3.3033	2.9888	0.7200	0.7122	1.09

*The entries highlighted correspond to games that are not violating the Sure Thing Principle. Expected θ corresponds to the quantum parameter that leads to the observed probability value in the experiment. Computed θ corresponds to the quantum parameter computed with the proposed heuristic*.

Small changes in the θ parameters can lead to a completely different probability outcomes. This has some relation with *deterministic chaos*, in which small differences in initial conditions yield widely diverging outcomes in a system. This chaos suggests how difficult the task of predicting human decisions is and how random it can be (Sterman, 1989).

### 7.2. Quantum Bayesian Network applied to the Two Stage Gambling Game

For the Two Stage Gambling Game, the overall results reported very small fit errors. The highest fit error percentage achieved was 16.3% and corresponds to the work of Kuhberger et al. (2001). Once again, the work of Kuhberger et al. (2001) is not showing a violation to the Sure Thing Principle, enhancing the previous conclusion that the proposed quantum-like Bayesian Network works best in situation where this violation exists.

**Figure 10:**
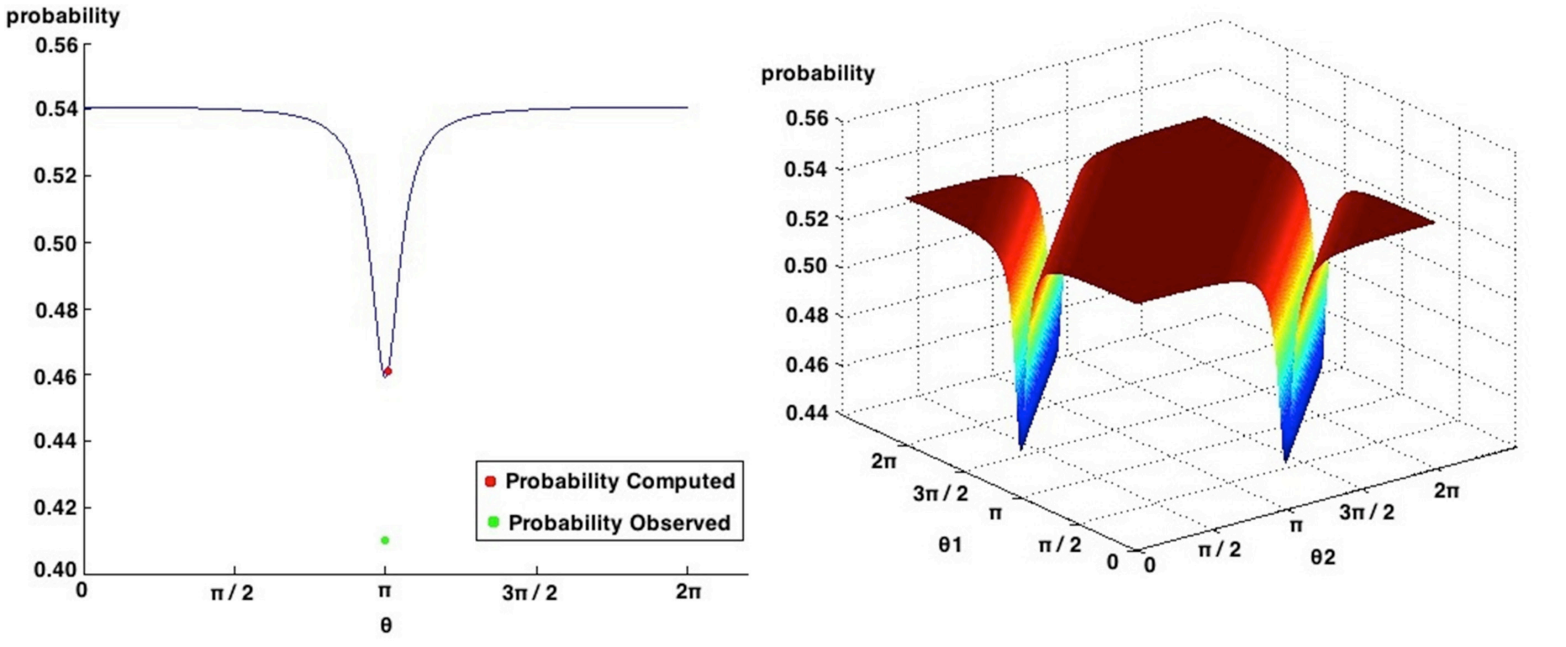
**Possible probabilities that can be obtained in the work of Lambdin and Burdsal (2007)**. The probabilities observed in their experiment and the one computed with the proposed quantum-like Bayesian Network are also represented.

In what concerns the work of Lambdin and Burdsal (2007) the proposed Quantum-Like Bayesian Network could not make accurate predictions. Figure 10 show all possible probabilities that can be obtained by varying the quantum parameters. As one can see, the minimum value that we can obtain corresponds to 0.4593. However, the observed probability reported by Lambdin and Burdsal (2007) corresponds to 0.41. This leads to a fit error of 12.02%.

In the work of Busemeyer et al. (2012), the authors applied the quantum dynamical model to reproduce the results obtained for the Two Stage Gambling Game and also explored the use of Hierarchical Bayesian methods to estimate the values of quantum parameters to simulate the player's *personal profile*: risk aversion, loss aversion, memory and choice. In the recent work of Busemeyer et al. (2015), the authors also compare the quantum model with a classical model using Bayes factor. They concluded that the quantum approach was preferred by the Bayes Factor.

### 7.3. Comparison with other works of the literature

In this section, we compare the results obtained with the proposed Quantum-Like Bayesian Network with the Quantum Prospect Decision Theory (Yukalov and Sornette, 2011). From all the analyzed models, this is the only one that can be called predictive due to its static heuristic: the Interference Quarter Law. The reason why we proposed a dynamic heuristic is because every decision problem is different and, consequently, quantum interference effects should also be different and not static. In the Quantum Prospect Decision Theory, the quantum interference term is fixed by the Interference Quarter Law, that is, the quantum interference term in the law of total probability is fixed to 0.25.

In the current model, since each decision problem is different, the proposed heuristic will compute a quantum θ parameter through similarities that the vector make between each other and these vectors are constructed from the experimental data. So, the vectors take into account the properties of each experiment, making it possible to compute different quantum interference terms for different decision problems.

Table 12 shows the results obtained for the Quantum Prospect Decision Theory and for the Quantum-Like Bayesian Network for the different works of the literature that tested violations to the Sure Thing Principle in the Prisoner's Dilemma Game and the Two Stage Gambling Game.

**Table 12:** **Comparison between the Quantum Prospect Decision Theory (QPDT) model and the proposed Quantum-Like Bayesian Network (QLBN) for different works of the literature reporting violations to the Sure Thing Principle**.

**Literature**	**Pr (Defect) Observed**	**Pr (Defect) Computed (QPDT)**	**Fit error (QPDT)**	**Pr (Defect) Computed (QLBN)**	**Fit error (QLBN)**
Shafir and Tversky, 1992	0.6300	0.6550	0.0397	0.6408	**0.0171**
Li and Taplin, 2002^b^	0.7200	0.5450	0.2431	0.7122	**0.0108**
Busemeyer et al., 2006a	0.6600	0.6250	**0.0531**	0.7995	0.2113
Hristova and Grinberg, 2008	0.8800	0.7000	0.2045	0.8968	**0.0191**
Tversky and Shafir, 1992	0.3700	0.3850	0.0405	0.3641	**0.0159**
Kuhberger et al., 2001	0.4800	0.3450	0.2813	0.4018	**0.1629**
Lambdin and Burdsal, 2007	0.4100	0.2900	0.2927	0.4085	**0.0037**
Average fit error	–	–	0.1651	–	**0.0630**

b *corresponds to the average of all seven experiments reported. The values in bold represent the models that obtained the lowest Fit error*.

In the end, the results from Table 12 demonstrate that, in general, the proposed Quantum-Like Bayesian Network together with the dynamic heuristic managed to fit the observed results in the several different experiments with an average fit error of 6.3%, whereas the Quantum Prospect Decision Theory achieved an average fit error of 16.51%.

One needs to take into account that in the Quantum Prospect Decision Theory and in the proposed Quantum-Like Bayesian Network, heuristics are used to estimate the quantum interference effects. This means that the heuristic can lead to a good fit of the data most of the times, but, in some cases, it can lead to completely wrong results. In the Quantum Prospect Theory, for instance, one can see the static Interference Quarter Law heuristic performed several estimations with big fit errors. The same is applied to the proposed Quantum-Like Bayesian Network. The difference is that this last model makes use of dynamic heuristics. Table 12 shows that the proposed dynamic heuristic overestimated the results in the works of Busemeyer et al. (2006a) and Kuhberger et al. (2001). This also happens due to the sensitivity of the θ parameters already discussed in Figure 9.

We also applied the Quantum Prospect Theory and the proposed Quantum-Like Bayesian Network to all experiments performed in the work of Li and Taplin (2002). Table 13 shows again great discrepancies between the average fit error obtained with the static heuristic of the Quantum Prospect Decision Theory. In general, the proposed model manages to fit all the different seven experiments with an average fit error of 6.41%, whereas the Quantum Prospect Decision Theory achieved an error of 24.23%. Most of the times, the Interference Quarter Law managed to produce lower estimations of the results observed during the several experiments. This shows that having a dynamical heuristic that is able to adapt to the different decision problems brings advantages in terms of predictive effectiveness.

**Table 13:** **Comparison between the Quantum Prospect Decision Theory (QPDT) model and the proposed Quantum-Like Bayesian Network (QLBN) for all the different experiments performed in the work of Li and Taplin (2002)**.

**Literature**	**Pr (Defect) Observed**	**Pr (Defect) Computed (QPDT)**	**Fit error (QPDT)**	**Pr (Defect) Computed (QLBN)**	**Fit error (QLBN)**
Game 1	0.6000	0.4502	0.2497	0.6313	**0.0522**
Game 2	0.6300	0.5333	0.1535	0.7011	**0.1129**
Game 3	0.8667	0.6334	0.2692	0.8113	**0.0639**
Game 4	0.7000	0.5667	0.1904	0.7341	**0.0487**
Game 5	0.7000	0.5333	0.2381	0.7006	**0.0009**
Game 6	0.8000	0.5500	0.3125	0.7169	**0.1039**
Game 7	0.7667	0.5500	0.2826	0.7159	**0.0663**
Average fit error	–	–	0.2423	–	**0.0641**

*The values in bold represent the models that obtained the lowest Fit error*.

## 8. Discussion and conclusion

In this work, we proposed an alternative quantum structure to perform quantum probabilistic inferences to accommodate the paradoxical findings of the Sure Thing Principle. We proposed a Quantum-Like Bayesian Network, which consists in replacing classical probabilities by quantum probability amplitudes. However, since this approach suffers from the problem of exponential growth of quantum parameters, we also proposed a similarity heuristic that automatically fits quantum parameters through vector similarities. This makes the proposed model general and predictive in contrast to the current state of the art models, which cannot be generalized for more complex decision scenarios and that only provide an explanatory nature for the observed paradoxes.

In Section 1.3, we established a set of research questions that we would like to address with the present research work. Their answers are detailed below.

1. Why do we need another quantum-like model to explain violations to the Sure Thing Principle?

Many of the models that have been proposed in the literature cannot be considered predictive. Most of these models require a set of quantum parameters to be fitted and, so far, the only way these models have to fit the parameters is to use the final outcome of the experiment to set the parameters in order to explain the experimental outcome. There is, however, one model in the literature that proposed a static heuristic to compute the quantum interference effects and can be called predictive. This model is the Quantum Prospect Decision Theory, proposed by Yukalov and Sornette (2011).

2. What is the advantage of the proposed approach? How can it make a difference toward the current well-established quantum models that have been proposed in the literature?

Since each decision problem is different, we believe that a quantum decision model would benefit from a dynamic heuristic that could take into account the decision problem's settings and come up with estimations for the quantum interference parameters. In the proposed model, quantum parameters are found based on the correlations that the vectors share between them. These correlations are explored through vector similarities that are computed using the Law of Cosines in a vector space. In this sense, we suggest that the quantum parameters that arise from interference effects might represent some degree of similarity between events. The previous work of Moreira and Wichert (2015) point out this semantic relation between vectors. In the end, the proposed model can be seen as a nonparametric method for estimating inference effects from a statistical point of view. It is a statistical model that is simpler than the previous Quantum Dynamical Model (Pothos and Busemeyer, 2009) and Quantum-Like Approach (Khrennikov, 2010) models proposed in the literature. The method makes use of the principles of Bayesian Networks, in order to obtain a more general and scalable model that can produce competitive results over the current state of the art models.

Experimental data demonstrated that the proposed heuristic managed to produce accurate fits to the data, overcoming the previously proposed Quantum Prospect Theory. This suggests that taking into account a dynamic estimation of quantum parameters is a good direction to build quantum-like predictive models.

### Conflict of interest statement

The authors declare that the research was conducted in the absence of any commercial or financial relationships that could be construed as a potential conflict of interest.
